# Vector competence and feeding-excretion behavior of *Triatoma rubrovaria* (Blanchard, 1843) (Hemiptera: Reduviidae) infected with *Trypanosoma cruzi* TcVI

**DOI:** 10.1371/journal.pntd.0008712

**Published:** 2020-09-24

**Authors:** Thaiane Verly, Stephanie Costa, Nathanielly Lima, Jacenir Mallet, Francisco Odêncio, Mirian Pereira, Carlos José de Carvalho Moreira, Constança Britto, Márcio G. Pavan

**Affiliations:** 1 Laboratório de Biologia Molecular e Doenças Endêmicas, Instituto Oswaldo Cruz/FIOCRUZ, Rio de Janeiro, Brazil; 2 Laboratório Interdisciplinar de Vigilância Entomológica em Diptera e Hemiptera, Instituto Oswaldo Cruz/FIOCRUZ, Rio de Janeiro, Brazil; 3 Universidade Iguaçu - UNIG, Rio de Janeiro, Brazil; 4 Laboratório de Ultraestrutura Celular, Instituto Oswaldo Cruz/FIOCRUZ, Rio de Janeiro, Brazil; 5 Laboratório de Doenças Parasitárias, Instituto Oswaldo Cruz/FIOCRUZ, Rio de Janeiro, Brazil; 6 Laboratório de Mosquitos Transmissores de Hematozoários, Instituto Oswaldo Cruz/FIOCRUZ, Rio de Janeiro, Brazil; University of California Davis, UNITED STATES

## Abstract

**Background:**

Several studies addressed changes on the insect vector behavior due to parasite infection, but little is known for triatomine bugs, vectors of *Trypanosoma cruzi*, the etiological agent of Chagas disease. We assessed infection rates and metacyclogenesis of *T*. *cruzi* (TcVI) in fifth-instar nymphs of *Triatoma rubrovaria* comparing with the primary vector *Triatoma infestans*. Also, biological parameters related to feeding-excretion behavior were evaluated aiming to identify which variables are most influenced by *T*. *cruzi* infection.

**Methodology/principal findings:**

Fifth-instar nymphs of *T*. *rubrovaria* and *T*. *infestans* were fed on mice infected with *T*. *cruzi* (TcVI). We compared the presence and the number of parasite evolutive forms in excreta of both triatomine species at 30, 60 and 90 days post-infection (dpi) with traditional statistical analyses. Moreover, both species were analyzed through generalized linear models and multinomial logistic regression hypotheses for seven behavioral parameters related to host-seeking and feeding-excretion. *Triatoma rubrovaria* and *T*. *infestans* had similar overall infection and metacyclogenesis rates of *T*. *cruzi* TcVI in laboratory conditions. Regarding vector behavior, we confirmed that the triatomine’s tendency is to move away from the bite region after a blood meal, probably to avoid being noticed by the vertebrate host. Interspecific differences were observed on the volume of blood ingested and on the proportion of individuals that excreted after the blood meal, revealing the higher feeding efficiency and dejection rates of *T*. *infestans*. The amount of ingested blood and the bite behavior of *T*. *rubrovaria* seems to be influenced by TcVI infection. Infected specimens tended to ingest ~25% more blood and to bite more the head of the host. Noteworthy, in two occasions, kleptohematophagy and coprophagy behaviors were also observed in *T*. *rubrovaria*.

**Conclusions/significance:**

Laboratory infections revealed similar rate of *T*. *cruzi* TcVI trypomatigotes in excreta of *T*. *rubrovaria* and *T*. *infestans*, one of the most epidemiological important vectors of *T*. *cruzi*. Therefore, TcVI DTU was able to complete its life cycle in *T*. *rubrovaria* under laboratory conditions, and this infection changed the feeding behavior of *T*. *rubrovaria*. Considering these results, *T*. *rubrovaria* must be kept under constant entomological surveillance in Rio Grande do Sul, Brazil.

## Author summary

Chagas disease is caused by the etiological agent *Trypanosoma cruzi* and is mainly transmitted through the excreta of triatomine vectors. It is endemic in Latin America and the Caribbean, affecting ~8 million people. Control programs are based on the elimination of domestic vectors through insecticide-spraying indoors, since there is no vaccine or efficient treatment for chronic patients. However, this strategy is not sustainable where native triatomine species are capable of colonizing peridomestic structures and reinvading human dwellings. Since the ‘virtual’ elimination of *Triatoma* *infestans* in Brazil (residual foci remain in Rio Grande do Sul and Bahia), specimens of the native *Triatoma rubrovaria* have been constantly collected inside human dwellings and peridomiciliary ecotopes in Rio Grande do Sul, southern Brazil, which might represent a real risk for human infections. Here we analyzed a unique and large dataset through classical and modern statistical methods to evaluate *T*. *cruzi* infection in *T*. *rubrovaria* and to identify host-seeking, and also feeding/excretion behavioral traits that could be influenced by the parasite. Our results indicated that the parasite infection caused changes in *T*. *rubrovaria* feeding behavior that could increase *T*. *cruzi* TcVI transmission. Moreover, this vector species had similar infection rate to one of the main important Chagas disease vectors in South America, *T*. *infestans*. Therefore, *T*. *rubrovaria* must be kept under constant entomological surveillance in Rio Grande do Sul, Brazil.

## Introduction

Vector-borne diseases represent nearly 17% of all known infectious diseases and are responsible for ~700,000 deaths annually [1]. Bloodsucking insects play the main role as pathogen transmitters (through their saliva or excreta) to humans. The incidence of a vector-borne disease is determined by several factors, including the density of primary vectors, efficient parasite replication and infectivity in vertebrate and invertebrate hosts, and the presence of susceptible hosts [2, 3].

The dispersion of parasites depends on the behavior of their arthropod vectors [4]. Pathogens can impose a fitness cost in life-history traits of vectors, which can influence parasite transmission [5, 6]. Most of the literature focuses on the costs of parasitism for mosquitoes (e.g. [7, 8]), and very little is known for triatomine bugs (Hemiptera, Reduviidae), vectors of *Trypanosoma cruzi* (Kinetoplastida: Trypanosomatidae), the etiological agent of Chagas disease (ChD).

ChD affects ~8 million people worldwide and is endemic in Latin America and the Caribbean [1]. Most patients during the acute phase are successful in treatment with trypanocidal compounds, but the treatment of chronic cases only reduces parasitic burden and does not prevent cardiac complications [9]. Therefore, control programs are based on the elimination of domestic vectors through insecticide-spraying indoors [10]. When entomological surveillance ceases, native vectors re-invade dwellings, which might cause a re-emergence of the transmission [11, 12].

The ‘virtual’ elimination of the primary vector *Triatoma infestans* (Klug, 1834) in Brazil (there are only residual foci in Bahia and the northwest of Rio Grande do Sul, Brazil) largely reduced the incidence and disease burden [12]. However, specimens of the native *Triatoma rubrovaria* (Blanchard, 1843) have constantly been collected inside human dwellings and peridomiciliary ecotopes in southern Brazil [13, 14]. Moreover, this species (i) has eclectic blood-feeding habitats, being capable of feeding on humans [15]; (ii) was already collected naturally-infected with *T*. *cruzi* [16]; (iii) excretes trypomastigotes (the infective form of *Trypanosoma cruzi* to humans) in laboratory conditions [17]; and also (iv) has bionomic characteristics that may be favorable to acquire the infection and to transmit the parasite to humans [18].

*Trypanosoma cruzi* is transmitted primarily through the urine/feces (excreta) of infected triatomines [19]. Therefore, host-seeking and probing, and feeding/excretion behaviors are some of the key parameters to determine their vectorial capacity [20]. Recent data show that this parasite causes negative impacts on vector fitness [21], such as impairing fertility, development and survival [22–26]. However, behavioral phenotypes of infected triatomines can be discrepant, with an increase in the locomotor activity of *Triatoma pallidipennis* [27] or a reduction in the activity of *Rhodnius prolixus* [4]. Considering feeding/excretion behavior, it seems that *T*. *cruzi* infection does not influence the amount of blood ingested by *R*. *prolixus* or the period of time between blood feeding and excretion [28], but it does for *T*. *infestans* [29]. Indeed, the impact of *T*. *cruzi* infection on the phenotype of insect behavior is understudied, requiring further attention [5].

Changes on vector behavior due to parasite infection may contribute to a better understanding of pathogen transmission dynamics. This study evaluates the infection rate and also the rate of metacyclogenesis of *T*. *cruzi* VI (isolated from field-collected *T*. *infestans*) in fifth-instar nymphs of *T*. *rubrovaria* specimens, comparing with the primary vector *T*. *infestans*. Moreover, it accesses the influence of *T*. *cruzi* infection on vector behavior parameters related to host-seeking and feeding-excretion.

## Methods

### Parasite culture

The *Trypanosoma cruzi* CL strain (TcVI) used in this study has derived from the excreta of a field-collected *T*. *infestans* from Rio Grande do Sul, southern Brazil, and was cultivated in Liver-Infusion Tryptose (LIT) medium, containing 10% fetal bovine serum (FBS) at 28°C [30]. Briefly, metacyclic trypomastigotes, harvested from LIT culture at the stationary phase, were used to infect Vero cells. Trypomastigote forms released from *T*. *cruzi*-infected Vero cells at four days post-infection (dpi) were used to infect Swiss Webster mice.

### Experimental hosts

Male Swiss Webster mice (18 to 20 g), provided by the Institute of Science and Technology in Bio-Models (ICTB/Fiocruz), were maintained in cages (19.56 x 30.91 x 13.34 cm, with four animals/cage) under controlled humidity and temperature (55% ± 5% and 22 ± 1°C, respectively) and light-dark cycles (12:12 h), with no food restriction. The animals were intraperitoneally infected with tissue-derived trypomastigotes CL strain (1x10^4^ parasites/0.1 mL NaCl 0.9%). The parasitemia was daily evaluated after 4 dpi through the Pizzi-Brener method [31]. When reaching parasitemia levels of 1 x 10^5^ parasites/mL, the animals were used to feed fifth-instar *T*. *rubrovaria* and *T*. *infestans* specimens.

### *T*. *rubrovaria* and *T*. *infestans* feeding and infection

Recently-merged fifth-instar nymphs of laboratory-colony reared *T*. *rubrovaria* (F3 generation) and *T*. *infestans* (F20 generation) were obtained from the *Laboratório Nacional e Internacional de Referência em Taxonomia de Triatomíneos*, Oswaldo Cruz Institute, Fiocruz, and maintained under controlled temperature and relative humidity (RH) conditions, at 26 ± 2°C and 70%, respectively. We chose this developmental stage due to low mortality rate [32], high feeding efficiency [33, 34] and easy handling. For the sake of comparison of infection and behavioral parameters obtained for *T*. *rubrovaria*, the primary vector *T*. *infestans* was included in the analysis, due to its high capacity for *T*. *cruzi* infection and transmission (positive control). As a negative control, we used non-infected *T*. *rubrovaria* specimens. Non-infected *T*. *infestans* with TcVI was not included in the analysis, since its bionomy and behavior are well established in the literature (*cf*. [5] and references there in [29]).

Ten days after molting to the 5th instar, insects were fed on anaesthetized Swiss Webster mice (combined ketamine hydrochloride 100 mg/kg and xylazine chloridrate 10 mg/kg), infected or not with *T*. *cruzi* CL strain (TcVI). Thirty days post-infection (30 dpi), starved triatomines exposed or not to TcVI were fed again on non-infected anaesthetized mice for behavioral analysis. For each experimental round, a single Swiss Webster mouse was laterally positioned in the presence of five triatomines in a rounded plastic container (12 cm diameter x 4 cm high) covered on the bottom with paper towels. Triatomine nymphs were centered in front of the Swiss Webster mouse’s abdomen 7 cm apart, and the blood source was offered for 30 minutes, including the adaptation time of the triatomine to the environment (i.e. the first 10-min period inside the container to recognize the host and start the blood meal, with no separation between the insects and the host). Specimens were replaced when not feeding after this period; notwithstanding, some experimental groups had less than five triatomines feeding on the mouse. The experiment was performed twice, under low artificial and indirect illumination at zeitgeber-time (ZT) 11, which corresponds to near the dusk, the peak of activity of triatomines for foraging [35]. The non-infective blood source was offered again at 60 and 90 dpi. One-hundred and twenty-seven *T*. *rubrovaria* specimens were used in behavioral analyses, which 58 were infected with *T*. *cruzi* (N_1_ = 25 and N_2_ = 33 for the first and second experiments, respectively) and 69 were not infected (N_1_ = 40 and N_2_ = 29), besides the 52 *T*. *cruzi*-infected *T*. *infestans* (N_1_ = 25 and N_2_ = 27).

Survived mice were euthanized after the experiments by peritoneal injection of ketamine hydrochloride (300 mg/kg) and xylazine chloridrate (30 mg/kg) in a final volume of 0.5 mL. Dead animals were frozen in a biological discard bag and sterilized at 121°C for 20 min for final disposal.

### Ethics statement

All animal procedures were approved by the Ethics Committee on Animal Use (CEUA) of Institute Oswaldo Cruz (IOC)/ Fiocruz (Licenses L015/17, LW-28/15 and L028/18).

### Detection of TcVI-infected *T*. *rubrovaria* and *T*. *infestans*

Non-infected (*T*. *rubrovaria*) and infected (*T*. *infestans*, as a positive control, and *T*. *rubrovaria*) groups were blood-fed at 30, 60 and 90 dpi. Triatomines’ excreta were individually-collected after blood feeding and spontaneous dejection in up to 4h. Twenty microliters of the excreta were then diluted in 100 μL of 1x phosphate-buffered saline solution (Thermo Fisher Scientific, Waltham, USA) for parasite counting in Neubauer chamber, using optical microscope Primo Star (Zeiss, Oberkochen, Germany) with a 400-X magnification. The morphological classification of *T*. *cruzi* evolutive forms as epimastigotes or trypomastigotes was based on cell shape, size, width, and motility (cf. [36] for further details). We defined as “transitory” all parasites that did not morphologically fit as either forms (*sensu* [37]). In the case of dubious morphology, we changed to a 1000-X magnification and classified according to the position of the kinetoplast in relation to the cell´s nucleus and by the emergence of the flagellum [36], whenever these structures were visible. In total, we analyzed the *T*. *cruzi* infection of 24 *T*. *rubrovaria* and 27 *T*. *infestans* specimens.

### Behavioral parameters

Specimens of negative control and TcVI-infected triatomine groups were analyzed individually at 30 dpi for the following parameters: (i) feeding time (min), which is the period between the first insertion of the proboscis into the mouse skin and its final removal (i.e. when insect moves away from the bite site); (ii) excretion time (up to 10 min) after feeding (min); (iii) excreta distance from the bite site (cm); (iv) volume of ingested blood (μL); (v) feeding efficiency (volume of ingested blood / feeding time; μL/min); (vi) excretion efficiency (feeding time / excreta distance; min/cm); and (vii) bite site on Swiss-mouse (head, dorse, tail, abdomen and legs). The amount of ingested blood was calculated through weighing specimens before and after the blood meal on a five-digit precision scale (AY220 model, Shimadzu Scientific Instruments, Kyoto, Japan) and converted to volume with a proportion of 1mg of additional weight after blood feeding ~ 1μL blood, since the mass of mouse blood is nearly equal to distilled water [38]. We also compared the proportion of individuals that excreted during the experiment between species (*T*. *rubrovaria* and *T*. *infestans*), with their status of infection (infected and non-infected), and also with the number of feeding attempts. We have considered a single attempt event when the vector inserted the proboscis into the mouse skin and then removed and walked away from the bite site.

### Statistical analysis

All statistical analyses were conducted in R environment [39] with proper packages. Infection and behavior data were not normally distributed (Shapiro-Wilk tests, p < 0.001) and, therefore, we used statistical tests that do not assume this probability distribution. The experimental replicates were not significantly different (non-significative Kruskal-Wallis and Pearson's Chi-squared Test for Count Data) and, therefore, were analyzed together.

#### Infection

Infections of *T*. *rubrovaria* and *T*. *infestans* with *T*. *cruzi* TcVI were analyzed both qualitatively and quantitatively. For each species, the presence/absence of infected triatomines at 30, 60 and 90 dpi, as well as the presence of trypomastigote, epimastigote and transitory *T*. *cruzi* forms in triatomine-infected excreta, were treated as dummy variables with binary data (qualitative analyses). Pairwise comparisons between *T*. *infestans* and *T*. *rubrovaria* were performed with Pearson's Chi-squared Test for Count Data (*X*^2^), including overall infection (i.e. regardless dpi and *T*. *cruzi* forms), and differences in the proportion of specimens with distinct parasite forms at the three dpi parameters analyzed.

Parasitic loads (quantitative analyses) were compared with the non-parametric Kruskal-Wallis (H) test, followed by Dunn’s multiple tests between 30, 60 and 90 dpi. Wilcoxon-Mann-Whitney W-test was also used to compare the parasite loads in *T*. *rubrovaria* and *T*. *infestans* for each timepoint. The Wilcoxon effect size of comparisons (r) was calculated with Rosenthal's formula [40]. The r value varies from 0 to 1, which 0–0.14 is considered as a small effect, 0.15–0.29 as moderate and 0.30 or higher as a large effect. Significance level was adjusted for multiple comparisons with the false discovery rate (FDR) method [41].

Generalized linear models (GLMs) with binomial distribution were performed with the “GGally” package [42] to identify significant effects of species and dpi (independent variables or predictors) on the presence of each of the different *T*. *cruzi* forms (dependent variable). The redundancy between predictor variables was accessed through the computation of the variance inflation factor (VIF) with the “car” package [43]. This factor measures how much the variance of a regression coefficient is inflated due to multicollinearity among predictors. Multicollinearity problems consist of including, in the same model, variables with similar predictive relationships with the outcome. VIF values are integer and always positive, and VIF < 5 represents absence of multicollinearity [44, 45]. Therefore, predictors with VIF > 5 were excluded from the analysis, as they would represent redundancy and may compromise model accuracy [44, 46]. Model selection was done through the calculation of second order Akaike’s information criterion scores (AICc) with the logit link function in the “AICcmodavg v.2.2” package [47]. Models were ranked and then compared with delta AICc (ΔAICc), for which ΔAICc > 2 indicated a clear model choice. Moreover, we calculated AICc model weights (Wt), which can be interpreted as the relative likelihood of a model, where Wt near 1.0 means most likely [48]. The strength of the association between each independent variable was expressed by the Odds Ratio (OR) with a 95% confidence interval (95% CI).

#### Behavior

The proportion of individuals that excreted during behavioral experiments was compared between species (*T*. *rubrovaria* and *T*. *infestans*) and infection status (infected and non-infected *T*. *rubrovaria*) through Pearson's Chi-squared Test for Count Data (*X*^2^). The same statistical method was used to compare the number of feeding attempts. Pairwise comparisons were performed with Wilcoxon-Mann-Whitney W-tests to analyze six behavioral parameters (i to vi, see *Behavioral parameters* section). Two different tests were performed: W1 –infected *T*. *rubrovaria* vs. infected *T*. *infestans*, to infer possible species differences; and W2 –non-infected and infected *T*. *rubrovaria*, to observe if *T*. *cruzi* TcVI infection altered any feeding-excretion parameter of this vector. Significance level was adjusted for multiple comparisons with the FDR method [41]. Also, we used the same statistical method to infer if the ingested blood volume was different for specimens that excreted and not excreted after the blood meal.

Generalized linear models (GLMs) assuming Gamma distribution were performed with the “GGally” package [42]. We tested the influence of the variables "species" (*T*. *rubrovaria* or *T*. *infestans*) and "infection" (infected or non-infected) on each estimated behavioral parameter (*cf*. Behavioral parameters section), which resulted in GLMs with seven different dependent variables. Moreover, behavioral traits were also included as independent variables in GLMs to test their effects on dependent variables (Table 1). Only behavioral traits that occurred before or during the dependent variable were included in the analyses as independent variables to avoid spurious associations between traits. For example, feeding time could explain the dependent variable blood volume as they occurred at the same time, but excreta distance could not explain the dependent variable "ingested blood volume", since the former variable is measured after the blood ingestion. All predictors were evaluated for multicollinearity through VIF, calculated with the “car” package [43]. Only non-redundant variables were maintained for model selection using second-order Akaike’s information criterion scores (AICc), as described above. Models were ranked and compared with ΔAICc and Wt. The strength of each parameter tested was expressed by the Odds Ratio (OR) with a 95% confidence interval (95% CI).

**Table 1:** Generalized Linear Models (GLMs) with Gamma distribution tested with different behavioral traits as dependent variables.

Dependent variables	Independent variables	N_M_	K_MAX_	K_BEST_	ΔAIC_C_
Feeding time	**Species**, infection, **ingested blood volume**	8	5	4	1.26
Excretion time	**Species**, infection, **ingested blood volume**, **excreta distance**, feeding time	32	7	5	1.15
Excreta distance	Species, **infection**, **ingested blood volume**, feeding time, **excretion time**	32	7	5	0.55
Ingested blood volume	**Species**, **infection**, **feeding time**	8	5	5	0.04
Feeding efficiency	**Species**, infection, **excreta distance**	8	5	4	2.33
Excretion efficiency	**Species**, infection, **blood volume**	8	5	4	0.62

The independent variables present in the best GLM are in bold. N_M_: number of GLMs tested; K_MAX_: maximum number of parameters tested in a single GLM; K_BEST_: number of parameters in the best GLM; ΔAIC_C_: delta Akaike Information Criteria corrected between the best and second-best GLM (*cf*. S3 Table for further details about tested GLMs).

The bite site on Swiss mouse of negative control (non-infected *T*. *rubrovaria*), tested (infected-*T*. *rubrovaria*) and positive control (infected *T*. *infestans*) groups were computed as categorical variables divided in five groups: head, tail, legs, dorse and abdomen. Multinomial logistic regression (MLR) was performed with the “nnet” package [49] to estimate the impact of predictor variables (bite sites) on infected and non-infected conditions. The significance of the regression coefficients was accessed after 2-tailed z-tests.

## Results

### *T*. *cruzi* infection

**Fig 1:**
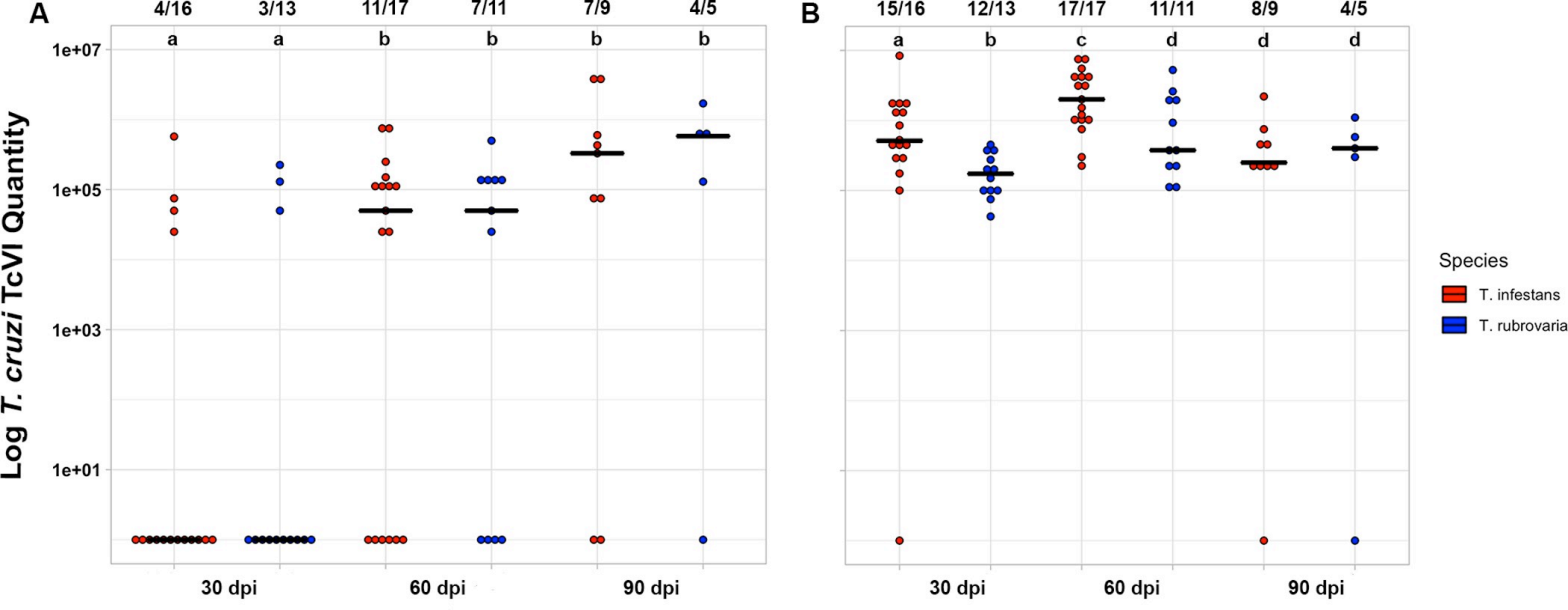
***T*. *cruzi* TcVI metacyclic trypomastigote (A) and epimastigote (B) loads in the excreta of *T*. *rubrovaria* and *T*. *infestans*.** The number of infected and the total number of specimens analyzed for each condition are shown above each dotplot column. Different lowercase letters on the top indicate statistically significant differences (Kruskal-Wallis-test; A: p (a and b) < 0.05; Wilcoxon-Mann-Whitney W-tests B: p (a and b) = 0.002 and p (c and d) = 0.02). dpi = days post infection.

Infection results revealed that both triatomine species had similar rates of *T*. *cruzi* TcVI infection, irrespective of the dpi or parasite form (*X*^2^ = 3.5 x 10^−31^, df = 1, p > 0.05). Twenty out of the 24 *T*. *rubrovaria* specimens fed on infected mice have excreted the parasite (83.3%), as well as 22 out of the 27 *T*. *infestans* (81.5%). The number of infected triatomines and the parasite load in the excreta were similar between species when trypomastigote and transitory forms of *T*. *cruzi* TcVI were considered (trypomastigote forms: *X*^2^ = 0.09, p = 0.77, and W = 0.03, p = 0.86; transitory forms: *X*^2^ = 3.27, p = 0.051, and W = 3.08, p = 0.054). However, the number of insects with metacyclic trypomastigotes increased in later days post infection, regardless the vector species (GLM: Trypomastigote ~ dpi, AICc = -91.57, with 72% probability of being the best model; S1 Table). The increasing number of trypomastigotes in infected excreta had strong positive association with time at 60 and 90 dpi (OR = 4.73 and 9.73, respectively; Table 2). The highest loads of metacyclic trypomastigote forms in triatomines' excreta were also found at these time points (H = 18.66, p < 0.0001; Dunn’s post-hoc 30-60dpi with p = 0.021 and 30-90dpi with p < 0.0001; Fig 1A).

**Table 2:** Generalized linear model for the presence of metacyclic trypomastigote forms of *T*. *cruzi* TcVI in the excreta of *T*. *rubrovaria* and *T*. *infestans* at different days post infection (dpi).

Parameters	OR	Estimate	SE	95% CI		P-value
(Intercept)	-	-0.97	0.41	-1.78	-0.15	0.02*
60 dpi	4.73	1.55	0.41	0.43	2.68	0.006**
90 dpi	9.73	2.26	2.26	0.75	3.78	0.003**

Note that 30 dpi is not shown, since it was used as the baseline condition. CI: Confidence interval; OR: odds ratio; SE: standard error.

* 0.01 < p < 0.05;

** 0.001 ≤ p ≤ 0.01.

Regarding the epimastigote forms, even though the GLM did not indicate differences between species or dpi for excreta infection rate (S2 Table), the amount of *T*. *cruzi* TcVI parasites in *T*. *infestans* excreta was higher than observed in *T*. *rubrovaria* at 30 and 60 dpi (W = 173 and 106, r = 0.56 and 0.41, with p = 0.002 and p = 0.02, respectively) (Fig 1B).

### Vector behavior

#### Pairwise comparisons

The weight of specimens before the experiments had non-significant differences when infection status (non-infected and infected *T*. *rubrovaria*) and species (*T*. *rubrovaria* and *T*. *infestans*) were compared (W-rank sum tests, p > 0.1). Therefore, there was no need to normalize the ingested blood volume data (median ± standard error 84.0±24.2, 102.5±14.7 and 109±16.39 mg, for non-infected and infected *T*. *rubrovaria* and infected *T*. *infestans*, respectively).

**Fig 2:**
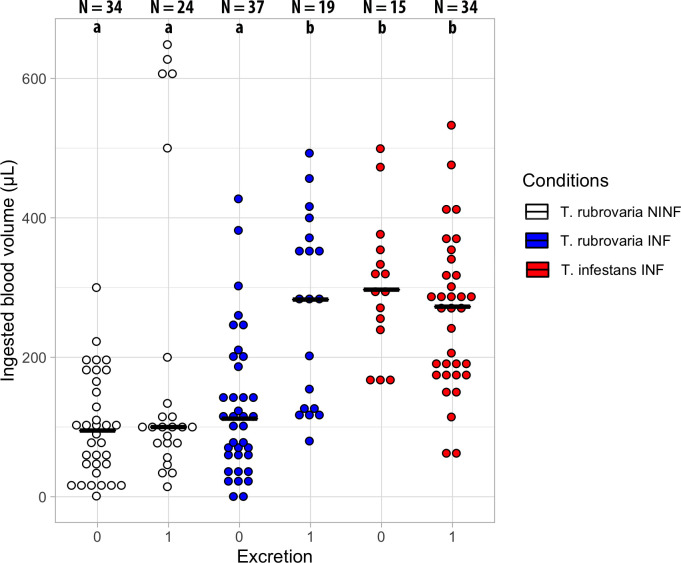
Ingested blood volume and excretion status (0: Not excreted, 1: Excreted) of non-infected *T*. *rubrovaria* (NINF), and infected- *T*. *rubrovaria* (INF) and *T*. *infestans* (INF). The number of samples analyzed for each condition is shown above each dotplot column. Different lowercase letters indicate statistically significant differences (Wilcoxon-Mann-Whitney W-test < 0.001).

The number of *T*. *infestans* individuals that excreted during behavioral experiments were proportionally higher than observed for *T*. *rubrovaria* nymphs (34/49 = 69.4% and 43/114 = 37.7%, respectively; *X*^2^ = 12.55, df = 1, p < 0.001). The infection status did not influence on the proportion of *T*. *rubrovaria* individuals that excreted up to 10-min after blood ingestion (24/58 = 41.4% and 19/56 = 33.9% for non-infected and infected specimens, respectively; *X*^2^ = 0.39, df = 1, p = 0.53). For *T*. *cruzi*-infected *T*. *infestans* and non-infected *T*. *rubrovaria*, there was no difference in the amount of blood ingested between individuals of the same species who excreted or not after the blood meal (W = 304.5, p = 0.29 and W = 332.5, p = 0.24); however, *T*. *cruzi*- infected *T*. *rubrovaria* individuals that have excreted after the blood meal ingested more blood than those that have not excreted (W = 133, p < 0.001; Fig 2).

**Fig 3:**
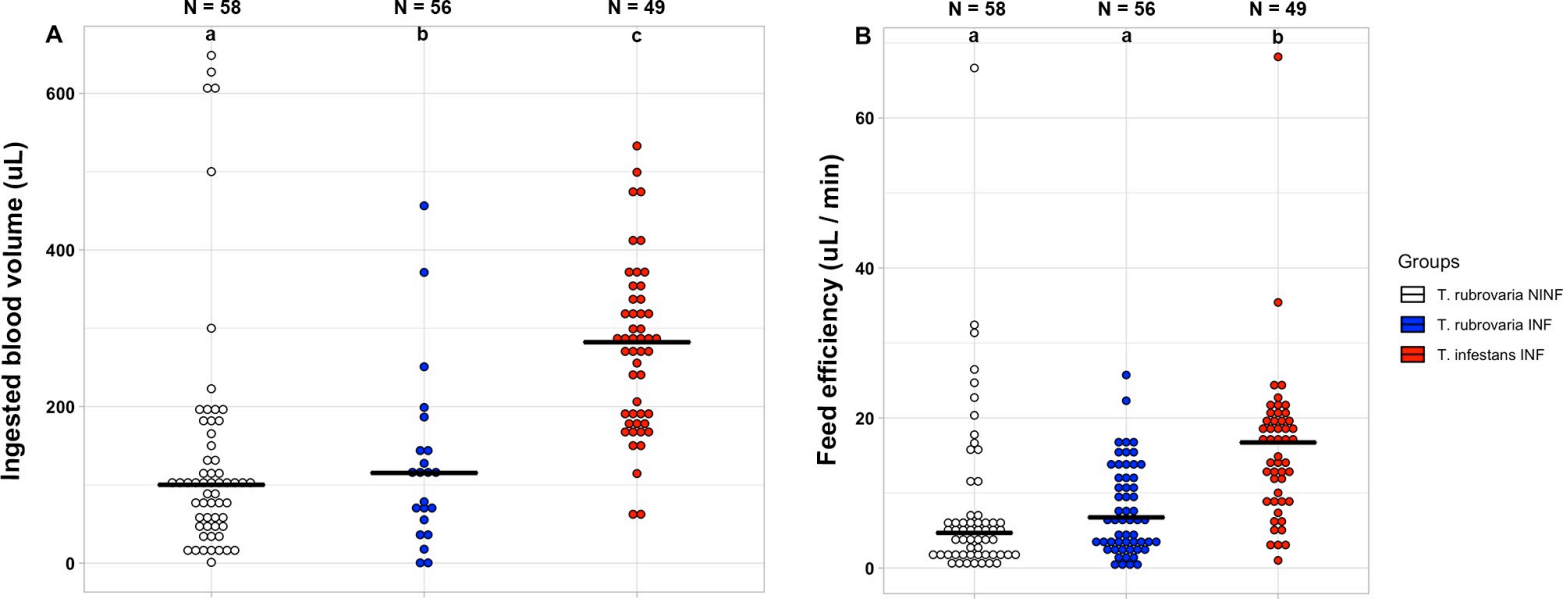
**Feeding parameters of non-infected *T*. *rubrovaria* (NINF), and infected *T*. *rubrovaria* (INF) and *T*. *infestans* (INF)–ingested blood volume (A) and feeding efficiency (B).** The number of samples analyzed for each condition is shown above each dotplot column. Different lowercase letters indicate statistically significant differences (Wilcoxon-Mann-Whitney W-test; **A**: p (ab) < 0.05, p (bc and ac) < 0.0001; **B**: p (ab) < 0.0001.

The number of feeding attempts was non-significant when species (*X*^2^ = 2.49, df = 1, p = 0.11) or infection status (*X*^2^ = 4.25, df = 2, p = 0.12) was compared, since the great majority of nymphs made a single attempt to feed on the blood of Swiss-mice (98.3%, 89.9% and 92.3% for non-infected and infected *T*. *rubrovaria*, and infected-*T*. *infestans*, respectively). The six feeding-excretion parameters were contrasted to assess possible behavioral differences between *T*. *rubrovaria* and *T*. *infestans* and to define what behavior aspects changed due to *T*. *cruzi* TcVI infection in *T*. *rubrovaria* (Table 3). Infected and non-infected *T*. *rubrovaria*, and also infected *T*. *infestans* had similar feeding time (median 95% CI: 15-28 min; W-tests, p > 0.05), excreted at a similar period after blood feeding (median 95% CI: 1-13 min; W-tests, p > 0.05) and at a similar distance from the bite (median 95% CI: 1-6cm; W-tests, p > 0.05), resulting in similar excretion efficiency (median 95% CI: 0.3–8.8 min/cm). Two behavioral parameters were different for the two triatomine species (Table 3 and Fig 3). Infected-*T*. *infestans* specimens ingested more blood than infected-*T*. *rubrovaria* (median 95% CIs: 239.4–301.3 μL and 113.8–198.8 μL, respectively; W = 733.5, p < 0.0001), and thus had higher feeding efficiency (median 95% CIs: 12.8–18.8 μL/min and 3.9–9.8 μL/min, respectively; W = 595, p < 0.0001). Although the median of feeding efficiency for infected-*T*. *rubrovaria* was slightly higher than for non-infected *T*. *rubrovaria* (6.8 against 4.7 μL/min), this difference was not statistically significant (W = 1388, p = 0.18). Interestingly, the ingestion of blood seemed to be influenced by *T*. *cruzi* TcVI infection, with higher ingestion of blood for *T*. *cruzi*-infected than for non-infected *T*. *rubrovaria* specimens (median 95% CIs: 113.8–198.8 μL and 75.0–105.3 μL, respectively; W = 1208, p = 0.016).

**Table 3:** Summary statistics and Wilcoxon-Mann-Whitney (W) tests between *T*. *cruzi* TcVI non-infected and infected *T*. *rubrovaria*, and infected-*T*. *infestans*.

Behavioral parameter	N	Median (95% CI)	W_1_	W_2_
Feeding time (min)	NNR = 58	20.5 (17.0–24.0)	1531	1850
	NIR = 69	23.9 (18.0–28.0)		
	NII = 52	19.0 (15.0–22.0)		
Ingested blood volume (μL)	NNR = 58	100.0 (75.0–105.3)	733.5***	1208*
	NIR = 56	126.5 (113.8–198.8)		
	NII = 49	282.2 (239.4–301.3)		
Excreta distance (cm)	NNR = 24	3.3 (1.5–6.0)	283.5	225.5
	NIR = 21	3.0 (1.0–5.5)		
	NII = 28	2.0 (2.0–3.5)		
Excretion time (min)	NNR = 27	5.0 (1.0–8.0)	268	281.5
	NIR = 21	3.0 (1.0–6.0)		
	NII = 36	6.0 (4.0–13.0)		
Feeding efficiency (μL/min)	NNR = 58	4.7 (3.3–5.8)	595***	1388
	NIR = 56	6.8 (3.9–9.8)		
	NII = 49	16.7 (12.8–18.8)		
Excretion efficiency (min/cm)	NNR = 24	1.2 (0.5–8.8)	291	184.5
	NIR = 21	0.8 (0.3–2.0)		
	NII = 28	0.7 (0.4–2.0)		

Statistically-significant results are highlighted in bold. N: number of samples; N_NR_: number of non-infected-*T*. *rubrovaria*; N_IR_: number of infected-*T*. *rubrovaria*; N_II_: number of infected-*T*. *infestans*; W_1_: W tests between infected *T*. *rubrovaria* and *T*. *infestans*; W_2_: W tests between infected- and non-infected-*T*. *rubrovaria*.

* 0.01 < p < 0.05;

*** p < 0.0001.

#### Generalized linear models

We fitted 96 GLMs to evaluate which feeding-excretion variables better explain the observed behavior phenotypes of *T*. *infestans* and *T*. *rubrovaria* (S3 Table). Three to four different models were comparable (ΔAICc < 2; cumulative Wt = 0.53–0.91) to explain most of the dependent variables, with the exception of “excretion efficiency”, in which two models were chosen (“species + blood volume” and “species” only, as independent variables; ΔAICc = 0.62; cumulative Wt = 0.53) and “feeding efficiency”, for which a single model was selected (“species + excreta distance” as independent variables; ΔAICc = 2.33, when compared to the second best model; Wt = 0.73). Hence, for the sake of clarity, we decided to show only GLM results for the model with the highest relative likelihood (Wt).

**Fig 4:**
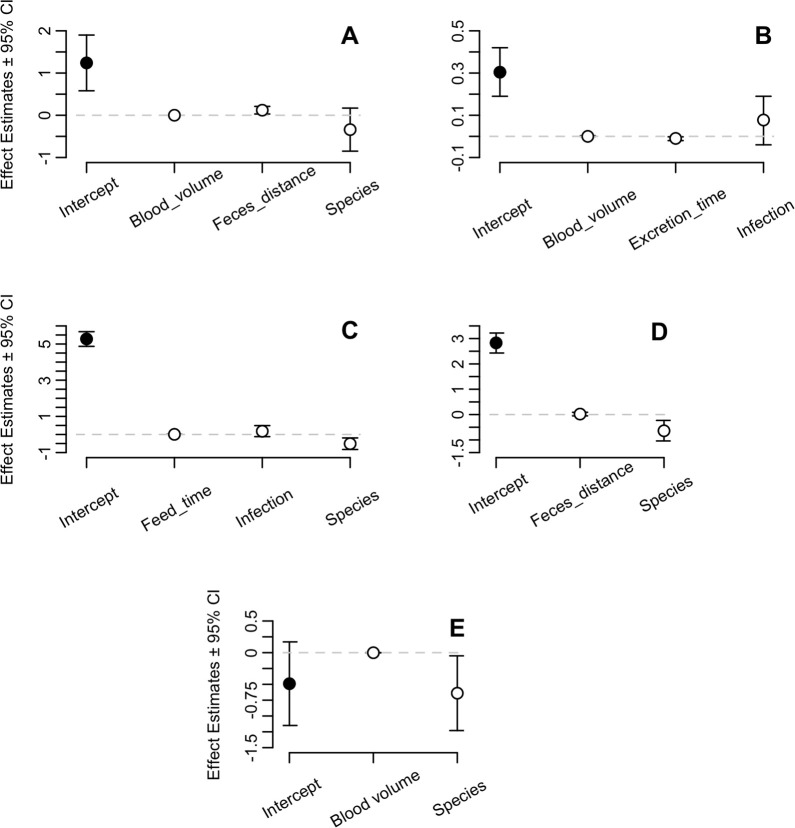
Model-averaged coefficients (± 95% CI) of the independent variables (white dots) and the intercepts (black dots) of generalized linear models. Excretion time after a blood meal (A), excreta distance from the bite (B), ingested blood volume (C), feeding efficiency (D) and excretion efficiency (E). Dashed line shows y-axis = 0. Colon marks between variable names mean “interacting with”.

The response variable “feeding time” could be explained by three different GLMs using as explanatory variables “species”, “infection” and “ingested blood volume”; however, none of the GLM coefficients were statistically significant (p > 0.05). When the dependent variable “excretion time” was analyzed, the independent variable “excreta distance” appeared in three out of the four GLMs with higher Wt (S3 Table) and was positively correlated (GLM coefficient 95% CI: 0.03, 0.21; p = 0.009) with mild causal influence (OR = 1.13; Table 4 and Fig 4). The opposite was also observed, with a significant correlation between the independent variable “excretion time” and the dependent variable “excreta distance” (GLM coefficient 95% CI: -0.02, -0.002; p = 0.017). However, since the GLM coefficient was near zero (i.e. between -0.1 and 0.1), there was no apparent causal influence on the dependent variable (OR = 0.99).

**Table 4:** Behavioral parameter results of the best generalized linear models with different dependent variables.

Variables	OR	Coefficient	SE	95% CI		P-value
**Excretion time ~ species + excreta distance + blood volume (N = 69)**						
Species (*T*. *rubrovaria*)	**0.71**	-0.34	0.26	-0.85	0.17	0.194
Excreta distance	**1.13**	0.12	0.05	0.03	0.21	0.009**
Blood volume	1.00	6.02E-04	7.78E-04	-9E-04	2E-03	0.438
Intercept	NA	1.24	0.34	0.58	1.90	4.5E-04***
**Excreta distance ~ infection + excretion time + blood volume (N = 69)**						
Infection	1.08	7.75E-02	5.82E-02	-0.04	0.19	0.188
Excretion time	0.99	-9.70E-03	3.93E-03	-0.02	-2E-03	0.017*
Blood volume	1.00	1.67E-05	1.60E-04	-3E-04	3E-03	0.917
Intercept	NA	3.04E-01	5.68E-02	0.19	0.42	1.2E-06***
**Blood volume ~ species + infection + feeding time (N = 163)**						
Species (*T*. *rubrovaria*)	**0.59**	-0.51	0.16	-0.83	-0.19	0.001**
Infection	**1.20**	0.18	0.15	-0.12	0.49	0.238
Feed time	1.01	6.75E-03	3.43E-03	3.2E-05	1.3E-02	0.051
Intercept	NA	5.28	0.21	4.87	5.68	2.0E-16***
**Feeding efficiency ~ species + excreta distance (N = 69)**						
Species (*T*. *rubrovaria*)	**0.52**	-0.64	0.21	-1.04	-0.23	0.002**
Excreta distance	1.02	0.02	0.04	-0.05	0.09	0.566
Intercept	NA	2.83	0.20	2.43	3.22	2.0E-16***
**Excretion efficiency ~ species + blood volume (N = 80)**						
Species (*T*. *rubrovaria*)	**0.53**	-0.64	0.30	-1.23	-0.05	0.037*
Blood volume	0.99	-1.03E-03	9.62E-04	-3E-03	8E-04	0.287
Intercept	NA	-0.49	0.34	-1.15	0.17	0.153

CI: Confidence interval; OR: odds ratio; SE: standard error; NA: not available.

* 0.01 < p < 0.05;

** 0.001 ≤ p ≤ 0.01;

*** p < 0.001. Colon marks between variable names mean “interacting with”. OR > 1.1 or OR < 0.9 are highlighted in bold.

*Triatoma rubrovaria* ingested less blood than *T*. *infestans* (GLM coefficient 95% CI: -0.83, -0.19; p = 0.001; OR = 0.59) and, consequently, had lower efficiency on blood feeding (GLM coefficient 95% CI: -1.04, -0.23; p = 0.002; OR = 0.52), besides having lower efficiency on excretion (GLM coefficient 95% CI: -1.23, -0.05; p = 0.037; OR = 0.53). Indeed, a negative impact of “species” (*T*. *rubrovaria*) on “excretion time” was observed (OR = 0.71), but GLM coefficient was not significant (95% CI: -0.85, 0.17; p = 0.194).

A positive impact of *T*. *rubrovaria* infection on blood volume was observed (OR = 1.20), which would mean that infected specimens of this species ingested more blood than non-infected ones. It is noteworthy, however, that the GLM coefficient was also not significant (95% CI: -0.12, 0.49, p = 0.238).

#### Multinomial logistic regression

**Fig 5:**
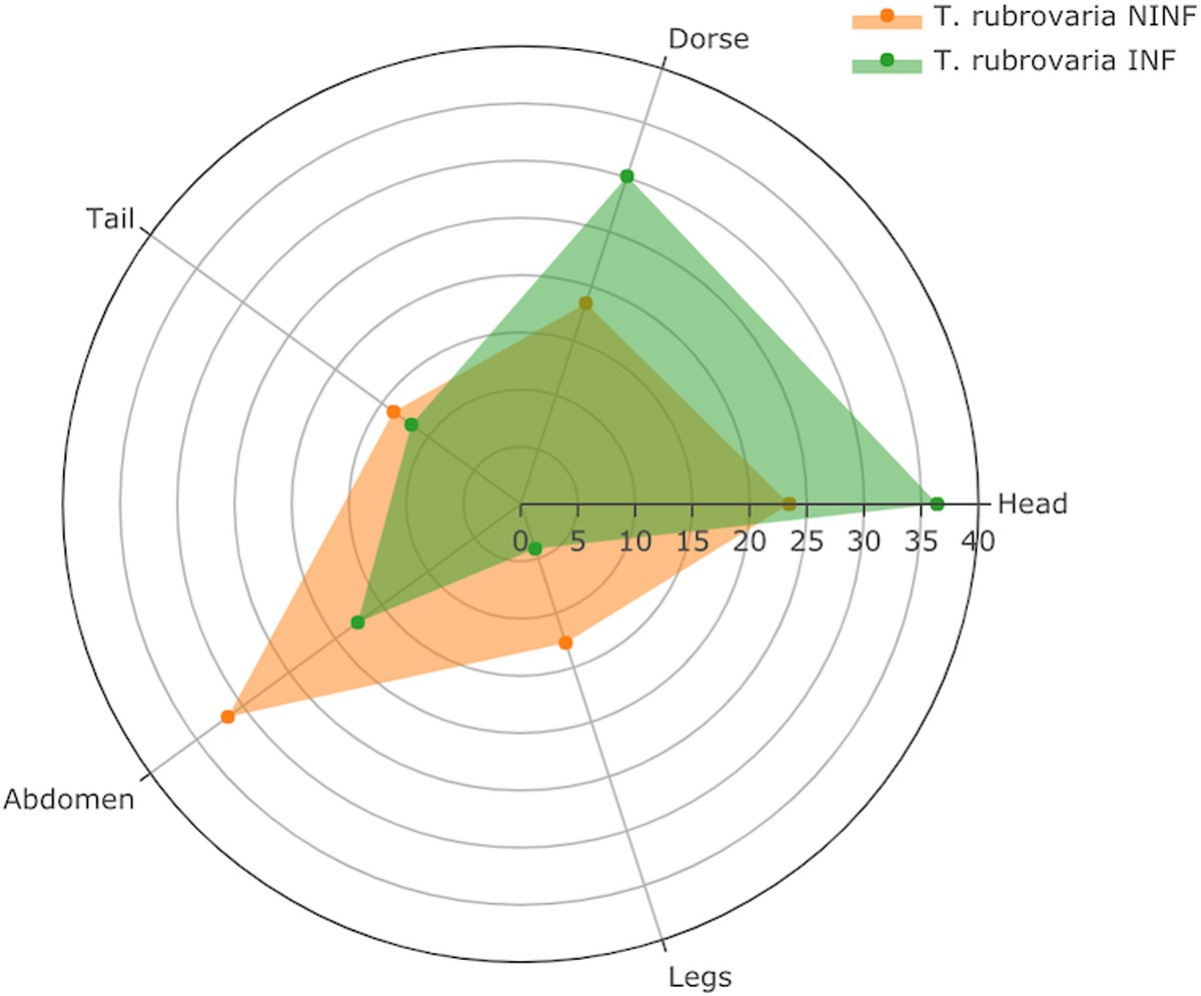
Radar chart exhibiting the percentage of bites of non-infected (NINF) and infected (INF) *T*. *rubrovaria* on different parts of mice’s body (head, dorse, tail, abdomen and legs).

Non-infected *T*. *rubrovaria* (N = 58) bit more frequent in the mice’s abdomen (31.6%), followed by the head (23.5%) and the dorse (18.4%). Infected-*T*. *rubrovaria* (N = 69) had a different behavior, biting more the head (36.4%) than the dorse (30.1%) or the abdomen (17.6%) (Fig 5).

The variables “species” and “infection” were tested in four different MLR models aiming to explain the dependent variable “bite site” (S4 Table). The best model considered only infection as an important predictor (MLR: bite site ~ infection; ΔAIC = 5.93 when compared to the second model; Wt = 90%). A strong and positive association was observed between *T*. *cruzi* infection and triatomine behavior of biting the head (OR = 2.94) and the dorse (OR = 2.80), but only the former was considered statistically significant in the model (MLR coefficient 1.08 ± 0.55, p = 0.03; Table 5). These results suggest that the bite behavior of *T*. *rubrovaria* specimens changed due to *T*. *cruzi* TcVI infection. Noteworthy, in two occasions, kleptohematophagy (an unfed individual ingesting 100.2 uL of blood and possibly hemolymph from another mouse-fed individual) and coprophagy behaviors (S1 Fig) were observed in *T*. *rubrovaria* fifth-instar nymphs.

**Table 5:** Parameter coefficients for multinomial logistic regression of bite sites.

Bite sites	Intercept Coefficient	Intercept SE	Infection Coefficient	Infection SE	P-value	OR
Dorse	-0.55	0.68	1.03	0.50	0.05	**2.80**
Head	-0.56	0.64	1.08	0.55	0.03*	**2.94**
Leg	-1.06	1.10	-0.55	0.78	0.48	0.58
Tail	-0.79	0.82	0.44	0.66	0.51	1.55

SE: standard error; OR: odds ratio.

* 0.01 < p < 0.05. In bold, OR > 2.

## Discussion

The Brazilian triatomine vector control campaign (1983–1988) and the successful Southern Cone Initiative involving other countries of South America (1991–2000) aimed the eradication of *T*. *infestans* through mapping and insecticide-spraying infested peridomiciles and dwellings [50, 51]. In southern Brazil, these strategies coupled with community-based vigilance achieved a sharp decline on domestic populations of this species, but also followed by an increase capture of *T*. *rubrovaria* nymphs and adults in anthropic ecotopes, suggesting that this species in fact begun to establish peridomestic and domestic populations only after the reduction of *T*. *infestans* populations [13, 14].

In this study, we aimed to evaluate some parameters related to vector competence of *T*. *rubrovaria* and to identify behavioral traits that could be influenced by *T*. *cruzi* infection. The results were compared with *T*. *infestans*, one of the primary vectors of *T*. *cruzi* in the Southern Cone of South America [52]. *Trypanosoma cruzi* infection (TcVI isolated from *T*. *infestans* in Rio Grande do Sul, Brazil) and metacyclogenesis rates, and also feeding-excretion parameters were evaluated in fifth-instar nymphs. We found compelling evidence that *T*. *infestans* and *T*. *rubrovaria* had similar *T*. *cruzi* infection rates, and also non-significant differences in most feeding-dejection parameters (feeding time, excretion time after blood meal, excreta distance from bite wound and, thus, excretion efficiency). Specimens of *T*. *infestans*, however, ingested more than twice the amount of blood that did *T*. *rubrovaria*, thus confirming higher feeding efficiency, as well as higher excretion rates after a blood meal. Our findings also suggested that *T*. *rubrovaria* individuals infected with *T*. *cruzi* TcVI ingested more blood than non-infected conspecifics (~25%) and also changed their biting behavior.

Metacyclogenesis is an *T*. *cruzi* adaptive differentiation due to nutritional and oxidative stress in the triatomine gut [53]. Therefore, it is unsurprising that vectors and parasites circulating at a same biotope may have a better interaction due to coevolution [54], including higher metacyclogenesis rates [55]. For triatomines and *T*. *cruzi*, this coevolution would result in successful infections and transmission cycles (i.e. presence of trypomastigote forms in insects’ excreta). Experimental infections of *Mepraia pallidipennis* from Morelos, Mexico, with two different strains of TcI–one from Morelos and another from Chilpancingo, Guerrero, Mexico, revealed that the parasite from the different geographic area (Chilpancingo) caused more debilitating effects on the vector, such as affecting their size and causing high parasitemia and low survival rate [24], thus evidencing possible role of genotype-by-genotype interactions [54] on triatomine populations and *T*. *cruzi* strains of the same geographical area. Hence, here we used a parasite (CL strain, TcVI) isolated from the excreta of field-captured *T*. *infestans* in Rio Grande do Sul, southern Brazil, same location where *T*. *infestans* and *T*. *rubrovaria* were collected, to ensure that the outcomes observed here were not caused by genotype impairments between vector population and parasite strain.

In this work, laboratory-infections of these species with *T*. *cruzi* TcVI revealed non-significant interspecific differences on the number of infected triatomines or on the parasite load in the excreta when trypomastigote and transitory forms were considered (Fig 1A). The number of triatomines with metacyclic trypomastigotes was significantly higher, as well as trypomastigote loads in the excreta in later days post infection (60 and 90 dpi), regardless the vector species (Table 2 and Fig 1). Therefore, our results warn about the great potential of *T*. *rubrovaria* to transmit *T*. *cruzi* TcVI. Interspecific differences were observed in Bolivia for three different vector species infected with *T*. *cruzi* TcVI (Tulahuén strain). Although the percentage of excreta with trypomastigote forms was similar for *T*. *infestans*, *T*. *sordida* and *T*. *guasayana* (sylvatic/peridomestic species in Bolivia), *T*. *infestans* had higher loads of trypomastigotes [56]. Regarding the epimastigote form, *T*. *infestans* presented here a higher number of this evolutive form at 30 and 60 dpi (Fig 1B). This result is possibly because that *T*. *infestans* ingested more blood than *T*. *rubrovaria* (Tables 3 and 4 and Fig 4) and heme (a pro-oxidant molecule released in large amounts upon hemoglobin degradation during blood digestion) seems to be responsible for epimastigote proliferation in a dose-dependent manner [57].

The effects of *T*. *cruzi* infection on triatomine behavior are scarcely investigated and the few studies available focused on fitness parameters, feeding-excretion behavior, aggregation/geotaxis and dispersion/locomotion (reviewed in [21],[58],[24],[29]). Feeding and excretion are behavioral parameters of paramount importance, since they determine the efficiency of *T*. *cruzi* transmission to vertebrate hosts [20]. In our study, non-infected and infected *T*. *rubrovaria*, and also infected *T*. *infestans* spent a similar period of time for feeding and excreting after the blood meal, and also they defecated at a similar distance from the bite. Previously, no difference was observed on the first two parameters between non-infected and infected *R*. *prolixus* collected in Venezuela with a *T*. *cruzi* TcI strain from the Amazon Basin. Nonetheless, discrepant results were observed for non-infected and infected *T*. *infestans* from Chaco, Argentina, with a strain of *T*. *cruzi* TcVI from Tulahuén, Chile. Infected specimens defecated earlier and in greater quantity than non-infected ones [29]. Earlier excretion was also observed for *Mepraia spinolai* (species responsible for *T*. *cruzi* transmission in arid and semiarid Chile) from Las Chinchillas National Reserve, Chile, infected with *T*. *cruzi* isolated from the same locality [59].

The amount of ingested blood seems to be positively influenced by *T*. *cruzi* TcVI infection, with a higher volume ingested by infected-*T*. *rubrovaria* (median 126.5 μL) than by uninfected specimens (median 100 μL) (Table 3). Moreover, *T*. *rubrovaria* specimens infected with *T*. *cruzi* that excreted during the experiments ingested twice the volume of blood when compared to non-infected *T*. *rubrovaria* or infected *T*. *rubrovaria* that did not defecate (Fig 2). The effect of infection on ingested blood volume was marginally observed in GLM, which even though the coefficient of infection was not significant, the odds ratio revealed a mild effect (Table 4). *Trypanosoma cruzi* did not affect the feeding time and probing in *R*. *prolixus* [28]. Botto-Mahan et al. [59] observed an increased biting rate in *M*. *spinolai* infected with *T*. *cruzi* compared to non-infected specimens, although the parasite did not interfere on the time interval until the first feeding attempt [59], or in the volume of ingested blood [60]. Estay-Olea et al. [61] observed for the same triatomine species that nutritional status of uninfected triatomines was higher than that from infected ones, strengthening the hypothesis that triatomines infected with *T*. *cruzi* need more blood for molting and excreting during/after a blood meal than uninfected individuals, probably to compensate the nutrients consumed by trypanosomes [61].

In close proximity, triatomines are capable of recognizing and being attracted by different host cues, such as host odorants and carbon dioxide, besides their extreme heat sensitivity due to the perception of heat and host-emitted infrared radiation (revised in [5]). In a recent study, third instar *T*. *pallidipennis* and *T*. *longipennis* infected with *T*. *cruzi* Chilpancingo isolate (TcI) were more attracted to compounds that mimic the odor released by the human face than non-infected insects [27]. These data are in agreement with our findings that infected *T*. *rubrovaria* sting more often in the head (36.4%) than in other body regions of the mice, whereas non-infected specimens bit more frequent in the mice’s abdomen (31.6%) (Fig 5). Different multinomial models were assayed for testing the variables “species” and “infection” as predictors of the bite site and the model that considers only infection as an important predictor was the most informative (S4 Table). Despite the multinomial logistic regression has showed a strong association between *T*. *cruzi* infection and triatomine behavior of biting the head (OR = 2.93) and the dorse (OR = 2.80), only the former was statistically significant in the model (Table 5). The resulting analyses suggest a change on the bite behavior of *T*. *rubrovaria* caused by *T*. *cruzi* TcVI infection.

Interspecific differences were observed regarding the proportion of individuals that excreted during the experiment, for which it was higher for *T*. *infestans* (Fig 2), and the volume of blood ingested, for which this species ingested more than twice of the blood volume of that observed for *T*. *rubrovaria* (Fig 3A). Consequently, the former species revealed higher feeding efficiency (Fig 3B). Indeed, the negative correlations between *T*. *rubrovaria* and the amount of blood ingested and blood uptake efficiency were considerable (Table 4 and Fig 4). Structural and physiological features were already associated with the feeding efficiency of triatomines. Blood suction, for instance, depends on the cibarial pump (a chamber structure with muscles in the head) that promotes a negative pressure, drawing blood up from inside the vessel. It is already observed that *T*. *infestans* is capable of ingesting higher amounts of blood per contraction of the cibarial pump when compared to *Triatoma brasiliensis* and *Triatoma pseudomaculata* [62]. Moreover, differences in the activity of vasodilatory and apyrase (platelet aggregation inhibitor) enzymes present in salivary glands [63, 64], and in hemagglutination and anticoagulant activities in the intestinal contents during feeding [65, 66] also influence blood meal size and thus the feeding efficiency of triatomines on vertebrate hosts. The higher ingestion rate of blood probably explains the shorter molting time of *T*. *infestans* over *T*. *rubrovaria* fifth-instar nymphs (64–83 and 110–131 days, respectively) [67, 68] and also a high population density of *T*. *infestans* inside human dwellings [69]. Since the amount of blood ingested is positively correlated with the volume of the excreta [70], it is also highly probable that *T*. *infestans* releases more parasites than *T*. *rubrovaria* during excretion.

The positive association observed in the GLM between the excreta distance from the bite wound and time of excretion after a blood meal (Table 4) is a commonsense feeding-excretion behavior of triatomines, but seldom tested. Our results here confirm that the triatomine’s tendency is to move away from the bite region after a blood meal, probably to avoid being noticed/predated by the vertebrate host, which reinforces the low probability estimates of direct transmission of *T*. *cruzi* from triatomines to humans [71]. *Triatoma* *rubrovaria* tended to excrete later than *T*. *infestans*; however, GLM coefficient was not statistically significant, probably due to the wide variance of measurements for this behavioral parameter. Another discrepant result between species was observed for the excretion efficiency parameter, which GLM results showed that *T*. *rubrovaria* is significantly less efficient (OR = 0.53), but W-tests were not significant (Tables 3 and 4). Our results differed from a previous work [56] which showed that *T*. *infestans* defecated during the blood meal at about five minutes before ending the blood ingestion. However, some differences in experimental settings should be highlighted, as follows: (i) we analyzed *T*. *infestans* infected with *T*. *cruzi*, while Loza-Murguia and Noireau [56] analyzed only non-infected specimens; and (ii) five triatomines were fed at the same time on a single host in this study (leading to some interruptions on blood feeding), while in the other work they individualized the vectors to feed on mice. Therefore, we raise the importance of including an epidemiologically-relevant species in experiments when analyzing a non-model species for comparison purposes.

Triatomines require numerous blood meals to complete their life cycle. Despite being rare [68, 72], coprophagy (the ingestion of stools), kleptohematophagy (blood draw from the gut of another triatomine) and hemolymphagy (to ingest hemolymph from other arthropods) have long been reported by several authors [73]. It has been observed *Triatoma circummaculata* and *T*. *rubrovaria* drawing hemolymph from wild cockroaches (*Blaptia dubia*) in natural rock piles [74, 75]. It remains unclear, however, if hemolymph as the only food source available is enough to complete their development cycle. In our work it is important to highlight in two occasions, an unfed nymph piercing the intestine of a recently fed nymph to obtain blood, besides having been observed in a single time a nymph feeding on the excreta of another individual. These behaviors were seen while the food source (Swiss mice) was being offered to the insects (S1 Fig). As far as we know, these are the first reports of coprophagy and kleptohematophagy in *T*. *rubrovaria*. Even with the availability of food source, the fifth-instar nymphs fed on the blood content of an engorged nymph. A recent hypothesis for this cannibalistic behavior is that the rapid intake of warm blood of the vertebrate host by the triatomine raises the insect's body temperature and consequently, leading to the attraction of unfed individuals to obtain food [76]. In an epidemiological context, the cannibalism could increase the possibility of ingesting the parasite and its further transmission from bug to bug [77].

This work has some caveats that must be bear in mind when interpreting our results. First, there will always be limitations in what the data generated in laboratory conditions can reveal about insect behavior in the field. Insects are submitted in the field to daily environmental oscillations of light, humidity and temperature, as well as vector aggregation that might shape different activity patterns from those observed in laboratory settings [58, 78, 79]. Moreover, laboratory-reared insects can lose the genetic variability observed in the field, probably due to inbreeding and stochastic processes such as founder effects and, thus, do not completely represent field populations [80]. Convincing evidence with experimental data supporting the impacts on behavior of rearing insects in laboratory conditions for many generations is scarce. Although there is a reduction in both body size and sexual dimorphism of laboratory-reared triatomines, which may reflect the high density of vectors in small containers and limited supply of blood meal [81], laboratory-reared populations of insects seem to exhibit similar performance to field populations. Ross et al. [82] observed only negligible changes in the development time, survival to adulthood, fecundity and egg hatch proportion when comparing *Aedes aegypti* populations from 2^nd^ and 22^nd^ generations. In triatomines, Ortiz et al. [83] observed that *R*. *prolixus* specimens maintained for over 30 years fed on chicken blood were attracted by human skin extracts in a similar way that F1 insects did. Experiments involving field (or recently field-caught) *T*. *rubrovaria* in natural or semi-natural conditions should be conducted to evaluate the impact of laboratory conditions on behavioral data.

Second, infections were confirmed and quantified through direct observations of trypanosomes in the excreta, and not through sensitive tools such as qPCR. Therefore, it is possible that we have detected as positive only triatomines with high infections. Although we believe that this limitation did not interfere the comparisons between *T*. *rubrovaria* and *T*. *infestans*, we probably excluded from the analyses individuals with low infections that could have different behavioral outcomes. Third, although we used a *T*. *cruzi* strain isolated from a *T*. *infestans* individual collected in the same locality of *T*. *rubrovaria* specimens used in this study, there is no evidence that *T*. *cruzi* TcVI lineage is infecting *T*. *rubrovaria* in the field. So far, only *T*. *rubrovaria* individuals infected with *T*. *cruzi* TcIII have been captured in the region [16, 84]. Entomological surveillance studies coupled with laboratory infections of *T*. *rubrovaria* with *T*. *cruzi* TcIII are needed to identify the *T*. *cruzi* strains circulating in this vector species and the possible effects of this parasite strain on feeding-excretion behavior.

Fourth, we analyzed feeding and excretion parameters in triatomines only at 30 dpi, when the number of vectors with trypomastigote forms in their excreta was significantly lower than observed at 60 and 90 dpi. Therefore, if behavioral changes observed here were a phenotypic outcome of the infection or a manipulation of triatomines by *T*. *cruzi* [27], we would expect to observe more significant behavior changes at 60 or 90 dpi. Molecular and histological studies are useful and needed approaches to understand the underlying mechanisms of possible behavioral manipulation [85]. Lastly, contradictory results regarding parasite infection influence on the triatomine behavior probably are associated to the variation in *T*. *cruzi* strain or genetic lineage used in infection assays and in vector species tested [86]. Moreover, differences in the experimental designs also may have an important contribution [29]. Therefore, there is an urgent need to standardize methodological conditions in future studies on behavioral traits of parasitized bugs to better understand the role of each type of *T*. *cruzi* on changes of vector behavior traits.

### Epidemiological relevance and concluding remarks

In conclusion, these results based on a unique and large dataset analyzed through classical statistical methods coupled with exhaustive generalized linear model and multinomial logistic regression hypotheses indicate that laboratory *T*. *cruzi* TcVI infection changed the *T*. *rubrovaria* feeding behavior. Infected individuals were capable of ingesting more blood than non-infected specimens and bit more often the host’s head. Despite the lower percentage of *T*. *rubrovaria* defecating after the blood meal and the lower volume of ingested blood when compared with one of the main important Chagas disease vectors in South America, *T*. *infestans*, these vector species shared similar overall infection and metacyclic trypomastigote rates of *T*. *cruzi* TcVI in their excreta under laboratory conditions, and also shared similar scores for most of the feeding-excretion parameters.

Lent and Wygodzinsky [20] highlighted that although most triatomines are potential vectors of *T*. *cruzi*, some conditions must be fulfilled to incriminate a species as ‘effective vector’ to humans, as follows: (i) wide geographical distribution, (ii) being capable of living in human habitations, (iii) anthropophilic behavior, and (iv) short interval between feeding and excretion. *Triatoma rubrovaria* is restricted to northern Argentina, Uruguay and southern Brazil [20] and although an increasing domiciliary and peridomiciliary invasion of *T*. *rubrovaria* [13, 14], there is no consistent evidence of intradomiciliary colonization, since only few nymphs have been captured inside dwellings [13]. This species has eclectic blood feeding habits, and a low percentage of its individuals was found with human blood content in the gut (1.3–8.0% [15, 87]). Together, these bionomic aspects emphasize the epidemiological findings that *T*. *infestans* had established larger colonies in southern Brazil and probably displaced *T*. *rubrovaria* from peridomiciles. However, the similarity between these species related to the overall infection and metacyclogenesis rates of *T*. *cruzi* TcVI in laboratory conditions and to the feeding-excretion time interval raises epidemiological concerns. The ‘virtual’ elimination of *T*. *infestans* probably triggered a recolonization process of human-modified habitats by the native species [14], as observed for other autochthonous species, such as *T*. *brasiliensis* and *Panstrongylus megistus* in northeastern and southeastern Brazil [69] and, thus, *T*. *rubrovaria* must be kept under constant entomological surveillance in Rio Grande do Sul, southern Brazil.

## Supporting information

S1 Fig Coprophagy. Fifth-instar nymphs of *Triatoma rubrovaria* feeding on another triatomine’s excreta.
(TIF)

S1 Table Competitive generalized linear models for the presence of metacyclic trypomastigote forms of *T*. *cruzi* TcVI in the excreta of *T*. *rubrovaria* and *T*. *infestans* at different days post infection (dpi). K: number of parameters; AICc: Akaike Information Criterion corrected; ΔAICc: difference between the AICc of a given model and the highest AICc, which ΔAICc > 2 means high support for a given model; Wt: model probability based on Akaike weight. Cum Wt: cumulative model probability based on Akaike weight.
(XLSX)

S2 Table Generalized linear model for the presence of epimastigote forms of *T*. *cruzi* TcVI in the excreta of *T*. *rubrovaria* and *T*. *infestans* at different days post infection (dpi). Note that 30 dpi is not shown, since it was used as the baseline condition. The colon mark (:) means interaction between variables. CI: Confidence interval; OR: odds ratio; SE: standard error. * 0.01 < p < 0.05; ** 0.001 ≤ p ≤ 0.01.
(XLSX)

S3 Table Competitive generalized linear models for six predicted feeding-excretion variables. AICc: Akaike Information Criterion corrected; ΔAICc: difference between the AICc of a given model and the highest AICc, which ΔAICc > 2 means high support for a given model; Wt: model probability based on Akaike weight; Cum Wt: cumulative model probability based on Akaike weight; LL: Log-likelihood of a given model. The models which ΔAICc < 2 are highlighted in bold.
(XLSX)

S4 Table Competitive multinomial logistic regression models for bite site parameters. AIC: Akaike Information Criterion; ΔAIC: difference between the AIC of a given model and the highest AIC, which ΔAIC > 2 means high support for a given model; Wt: model probability based on Akaike weight; Cum Wt: cumulative model probability based on Akaike weight.
(XLSX)
