# Supplementary figures and images for: Vector competence and feeding-excretion behavior of Triatoma rubrovaria (Blanchard, 1843) (Hemiptera: Reduviidae) infected with Trypanosoma cruzi TcVI
Source: PLoS Negl Trop Dis. 2020 Sep 24;14(9):e0008712. doi: 10.1371/journal.pntd.0008712 (PMC7544132; doi:10.1371/journal.pntd.0008712)

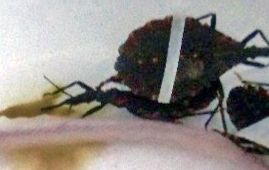

Supplement: S1 Fig — Fifth-instar nymphs of Triatoma rubrovaria feeding on another triatomine’s excreta. (TIF) [file pntd.0008712.s001.tif]
